# Multivesicular hepatic human hydatid cyst from Iran: First genotyping‐based confirmation

**DOI:** 10.1002/ccr3.5336

**Published:** 2022-02-03

**Authors:** Eissa Soleymani, Sohrab Sayyadi, Hossein Pazoki, Mahdi Fakhar, Elham Sadat Banimostafavi, Mohsen Kolivand, Lotfollah Davoodi, Mostafa Soleymani

**Affiliations:** ^1^ Department of Parasitology and Mycology Student Research Committee Hamadan University of Medical Sciences Hamadan Iran; ^2^ 92948 Department of Surgery Iranian National Registry Centre for Hydatid Cyst, Razi and Imam Khomeini Hospitals Mazandaran University of Medical Sciences Sari Iran; ^3^ Student Research Committee School of Medicine Shahid Beheshti University of Medical Sciences Tehran Iran; ^4^ 92948 Toxoplasmosis Research Centre Communicable Diseases Institute Iranian National Registry Centre for Lophomoniasis and Toxoplasmosis Mazandaran University of Medical Sciences Sari Iran; ^5^ 92948 Department of Radiology Toxoplasmosis Research Centre Communicable Diseases Institute Imam Khomeini Hospital Mazandaran University of Medical Sciences Sari Iran; ^6^ Department of Infectious Diseases Antimicrobial Resistance Research Center Faculty of Medicine Mazandaran University of Medical Sciences Sari Iran

**Keywords:** *Echinococcus granulosus*, genotyping, hydatid disease, liver, multivesicular cyst

## Abstract

Cystic echinococcosis is one of the most important zoonotic parasitic diseases caused by the tapeworm *Echinococcus granulosus*. To date, the genotype of multivesicular CE has not been identified. In this regard, the genotyping of multivesicular types of CE could help clinicians understand and manage the disease effectively.

## Graphical abstract

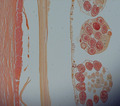

## 1 INTRODUCTION

Iran is a hyperendemic region for *Echinococcus granulosus* (*E*. *granulosus*) and hydatid disease. Here, we report a case of hepatic multivesicular hydatid cyst with symptoms of weakness and anorexia. RFLP‐PCR was used to confirm the diagnosis and *E*. *granulosus* typing, and it was determined that *E*. *granulosus* belongs to G1 genotype.

Cystic echinococcosis (CE) is one of the most important zoonotic parasitic diseases caused by the tapeworm *E*.* granulosus*. Investigations in various parts of Iran confirm that Iran is a hyperendemic area for *E*. *granulosus*.^1^ CE is one of the most critical zoological diseases in northern Iran, and the provinces of Mazandaran, Gilan, and Golestan are known as endemic areas for this disease. According to reports, the prevalence of this disease in northern Iran is on average 9%, which is one of the most polluted areas in Iran.^2^ Domestic dogs, as definitive hosts of the adult *E*. *granulosus*, play the most important role in scattering infection in Middle Eastern countries, including Iran, via contamination of the environment. Additionally, sheep, goats, cattle, camels, and buffaloes have been repeatedly found infected with CE in Iran.^3^ Molecular data show that the G6 genotype (camel/dog strain) and G1 genotype (common sheep strain) of *E*. *granulosus* mostly occur in Iran.^5^ CE is usually located in the liver and lung, but several studies have been reported from unusual sites such as the appendix, spleen, spinal, orbit, ovary, parotid glands, pancreas, skin, lymphatic glands, uterus, and tonsils.^6^ The right lobe of the liver and the lungs are the usual locations for involvement, whereas the left lobe of the liver is relatively rarely involved.^7^

The important risk factors in the life cycle of *E*. *granulosus* include contact with dogs and sheep, eating vegetables, and geophagy as a potential source in Iran.^6^ In Iran, the rate of human CE infection is 5%, and Mazandaran Province, in northern Iran, is one of the endemic areas for this parasite.^2^, ^4^ So far, the genotype of multivesicular CE has not been reported anywhere in the world, and the most related reports are limited to pathological confirmation of the CE. Here, we present an unusual case of a multivesicular hepatic CE from northern Iran.

## 2 CASE PRESENTATION

In this report, the case was reported according to the consensus‐based clinical case report (CARE) guidelines.^8^ In July 2013, a 34‐year‐old woman from Mazandaran Province in northern Iran was referred to the teaching Razi Hospital in the rural area of Qaemshahr town with a month's worth of LUQ (left upper quadrant) pain, loss of appetite, and malaise. On physical examination, blood pressure was 110/70 mmHg and the heart rate was 120 bpm. All routine blood tests were normal (SGOT: 95, SGPT: 79, Alkaline phosphatase: 138, WBC: 7.8 × 10^3^ µ/L, RBC: 3.78 × 10^6^ µ/L, HGB: 11.1 g/L, HCT: 33.2%, MCV: 87.8 fL, MCH: 29.4 pg, MCHC: 33.4 g/dL, Platelet: 241 × 10^3^ µ/L) except for a little eosinophilia. A multilobular cystic lesion with measurements of 12 × 8 cm fills nearly all the left lobe of the liver. On sonography, cystic lesions with 4 × 2 × 7 cm in dimensions on the left lobe of the liver were reported.

**FIGURE 1:**
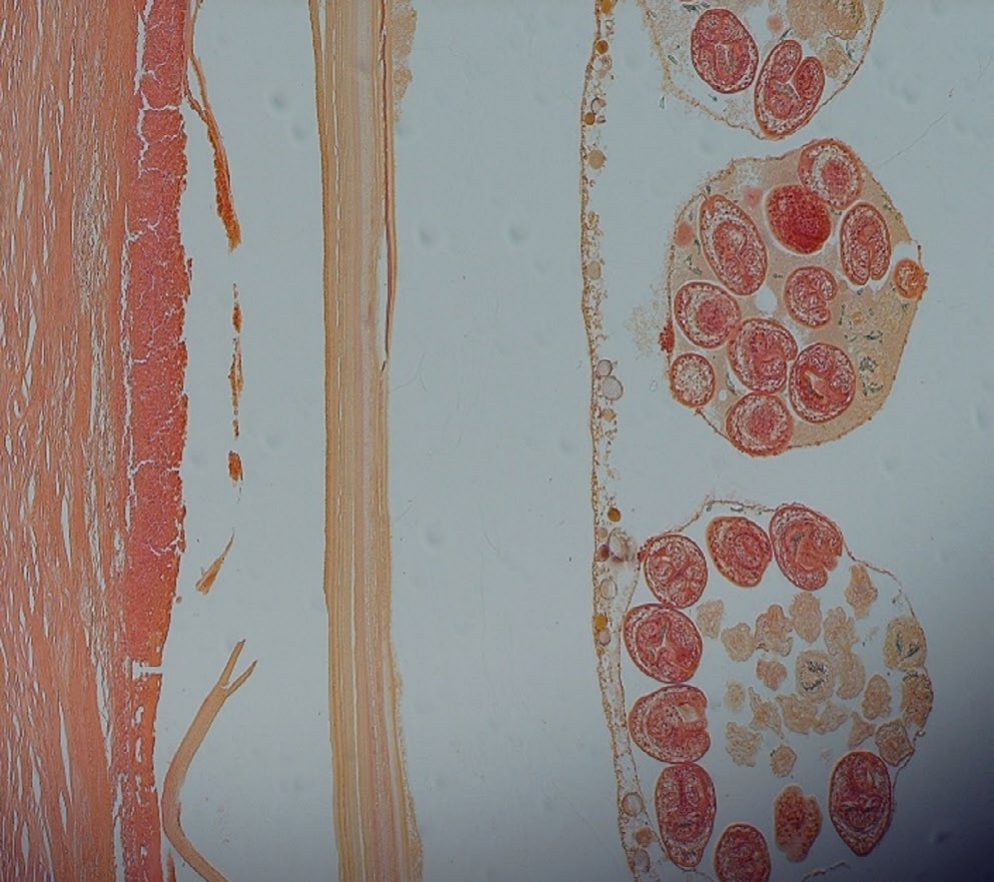
Cross‐section of laminated and germinal layers of the hepatic multivesicular cyst. (H&E staining) ×400

**FIGURE 2:**
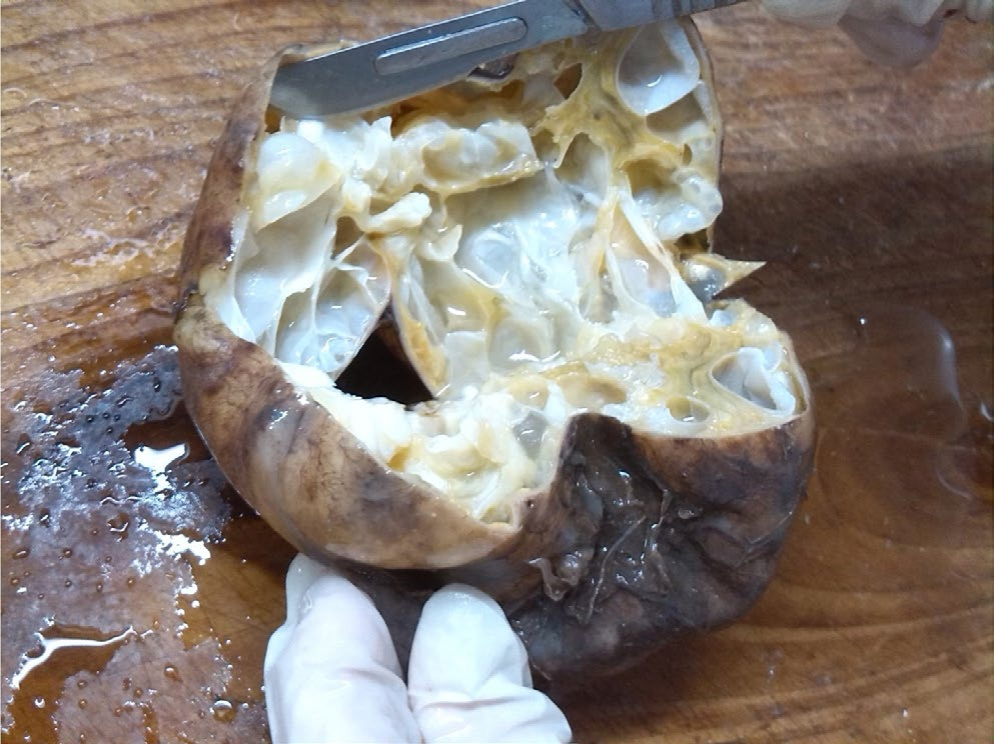
Hepatic multivesicular (honey comb) cyst removed from the patient in the North of Iran

The pathological investigation showed laminated and germinal layers of *Echinococus* spp. (Figure 1). Additionally, fine needle aspiration (FNA) also revealed many protoscolexes (hydatid sand). Surprisingly, gross pathology demonstrates unusual cysts as unilocular and multivesicular CE. Therefore, pathological examinations confirm hydatidosis in the patient. After cystotomy, numerous daughter cysts ranging from 2 to 30 mm were observed. The daughter's cysts were carefully removed (Figure 2).

Germinal layers in hepatic sections were found to be yellowish and smaller in diameter than a laminated layer. The pathological investigations revealed the lumen includes brood capsules containing multiple protoscolices (H & E, ×400) accompanied by necrosis and infiltration of inflammatory cells, predominantly eosinophils. In the cross‐section of cyst germinal and laminated layers, booklets, calcareous corpuscles, and brood capsule are considered diagnostic keys.^6^

Moreover, for DNA extraction, protoscoleces and germinal layers were removed from the cyst and washed two to three times with normal saline. Two hundred microliters of laser slip was added to the sample. The sample was melted and frozen five times with liquid nitrogen and a hot water bath. Proteinase K (10 μl) was added and incubated at 37°C for 24 h. Then, 100 microliters of phenol/chloroform was added to it and centrifuged at 16,500 *g* for 5 min. We transferred the upper phase to another micro‐type, and the same volume of cold absolute ethanol was added, to deposit DNA at the bottom of the micro‐type. Then, we centrifuged the sample for 5 min at 16,000 *g*, and the upper liquid was discarded. Additionally, after drying ethanol, 100 ml of sterile distilled water was added and stored at 4°C until used for PCR.

The specific RFLP‐PCR method is performed by a fragment of the mitochondrial cytochrome c oxidase subunit 1 (cox1) gene containing 100 ng of DNA was performed as follows: 20 pmol of each primer, 1×Taq DNA polymerase buffer (20 mM Tris–HCl, pH 8.4, and 30 mM KCl, 0.04 mM dNTP mix, 1.5 mM MgCl2) and 1.25 U Taq DNA polymerase in a final volume of 25 μl. This was amplified using a specific primer pair, including JB3 (5′TTTTTTGGGCATCCTGAGGTTTAT‐3′) and JB4.5 (5′TAAAGAAAGAACATAATGAAAATG‐3′).^9^

In the next step, the samples were amplified as follows: initial denaturation at 95°C for 5 min and 35 cycles at 95°C for 45 s, 52°C for 30 s (annealing), and 72°C for 45 s (extension), and this was followed by a final extension cycle at 72°C for 10 min.^10^ Finally, 10 μl of each amplified product was analyzed by electrophoresis on a 2.5% agarose gel and stained with SYBR green. The PCR product bands were observed under UV light and sized by measuring them with a 100‐bp DNA ladder, and the Cox1 gene was successfully amplified for this isolate. The PCR amplification is part of the cox1 (pcox1) gene, an expected 446‐bp fragment for the CEs DNA sample.

All PCR products were digested using the HpaII restriction enzyme for COX1 by the company's recommended buffer (ThermoS Scientific) at a final volume of 20 μl. The restricted fragments on the 2.5% agarose gel were separated by electrophoresis, dyed using SYBR Green, and observed under ultraviolet radiation. The *E*. *granulosus* isolate was tested by PCR‐RFLP evaluation of COX1 using HpaII restriction endonuclease enzyme. PCR‐RFLP analysis with HpaII digestion showed 2 bands of 304 and 140 bp samples, and the pattern of the PCR enzyme digestion band with HpaII showed the G1 genotype. Thus, the liver cyst belonged to the G1 genotype.

The cyst space was placated following a wash with hypertonic saline (3% NaCL). The patient was discharged on the 8th day post‐operation without any complications. After 1 year of operation and treatment (100‐mg mebendazole, an oral dose), the patient was free of symptoms and no problem was reported.

## 3 DISCUSSION

The first account of the CE related to the time of Hippocrates, and the first successful surgical excision to remove the CE was performed by Long in 1932.^11^, ^12^ There are many theories about how to form a daughter cyst, but the most important of them is trauma. If the cyst does not have a daughter cyst, it is called ‘univesicular’ and if it has one or more daughter cysts, it is called ‘multivesicular’. CEs are usually at the beginning of the infection and grow slowly in the soft tissues. After that, the daughter cysts then form multiple vesicles (daughter cysts) within the mother's large cyst. Also, the main treatment for the CEs is surgical excision, which is accompanied by drug treatment after surgery.^13^

Hydatidosis is asymptomatic in most cases and is usually diagnosed by imaging tests that the person has had for another reason.^14^ Serological tests have many false‐negative results and are unreliable. They are also negative in many cases of CE, but imaging techniques such as CT scans and ultrasonography have the fewest false‐negative results. CT scans also look good for displaying density, morphology, and the extent of these lesions, and in most cases, they accurately determine the exact relationship of the lesion to adjacent structures. These imaging methods are considered as a reliable and avilable choice for CEs for diagnosis preoperative, even in unusual places.^11^, ^15^

Although it was observed in the pathological observations of the protoscolex, the cyst was similar to the polycystic cyst, both in ultrasound and pathology, and our confirmation test was for an accurate PCR diagnosis. Although PCR is not commercially available in all laboratories and is usually used for research purposes only,^16^ but in cases where there is a possibility of error in the diagnosis of unusual forms of CE, it is recommended to use the PCR diagnostic method to prevent incorrect treatment. We also recommend that by using both morphological and molecular approaches together, more accurate and complete information can be obtained to better diagnose this parasite.

Our case is the first multivesicular report in Mazandaran province whose genotype (G1) has been determined. In Iran, several genotypes of the *E*. *granulosus* parasite have been identified, but the most prominent of these is the G1 genotype. The G1 genotype can be found in sheep, goats, cows, camels, and humans. Studies have also shown that there are different subgroups of G1 in Iran.^17^ Furthermore, previous research has shown that G1 is the most common genotype in Mazandaran province, and our findings are consistent with previous studies.^18^, ^19^ These results show that the dog and sheep cycle is the most common route of transmission of this parasite in Mazandaran province. The dominance of the G1 genotype in northern Iran affects the relationship between the human population, sheep, and cattle, and, considering that Mazandaran province has suitable pastures for traditional breeding of domestic animals and the existence of stray dogs in this area, it could be a potential reservoir for human pollution. In addition to early and correct diagnosis, to prevent parasitic infections, health standards must be raised and implemented in this area. Following up on patients, eradicating stray dogs, and providing health education to the people of the region should be included in the training programs to reduce the prevalence of CEs.

## 4 CONCLUSION

As a whole, the genotype of multivesicular CE has not been identified yet, and all reported cases are mostly identified based on parasitological/pathological features. In this regard, the genotyping of multivesicular types of CE could help clinicians understand and manage the disease effectively. To the best of our knowledge, this is the first case of a multivesicular hepatic hydatid cyst whose genotype has been determined.

## CONFLICT OF INTEREST

None declared.

## AUTHOR CONTRIBUTIONS

ES and LD contributed conception and design of the study. ES wrote the first draft of the manuscript. MF, ESB, LD, MK, and MS wrote sections of the manuscript. SS and HP extracted data from the patient's sheets. MS was responsible for collecting data and submitting the manuscript. All authors contributed to critical manuscript revision, read and approved the submitted version.

## ETHICAL APPROVAL

This research was reviewed and approved by the research ethics committee of Mazandaran University of Medical Sciences (IR.MAZUMS.REC.1397.419).

## CONSENT

The patient wrote the informed consent for participating in this study. Written informed consent for the publication of this case report was taken from the patient.

## HUMAN AND ANIMAL RIGHTS

This study was conducted according to the Declaration of Helsinki principles. Also, CARE guidelines and methodology have been followed in this study.

## Data Availability

The data are available with the correspondence author and can be achieved on request.
